# Mobile apps for psychotic disorders: a systematic review protocol

**DOI:** 10.1186/s13643-025-02943-8

**Published:** 2026-01-09

**Authors:** Ananya Ananthakrishnan, Aditya Narain Sharma, David Anderson, Rohit Shankar, Edward Meinert

**Affiliations:** 1 Translational and Clinical Research Institute, Newcastle University, Newcastle, UK; 2 Specialist Adolescent Mood Disorders Service (SAMS), Tyne and Wear NHS Foundation Trust, CumbriaNewcastle Upon Tyne, Northumberland UK; 3 Academic Psychiatry, Translational and Clinical Research Institute, Newcastle University, Newcastle Upon Tyne, UK; 4 Peninsula Medical School, Faculty of Health, University of Plymouth, Plymouth, PL4 8AA UK; 5 Cornwall Partnership NHS Foundation Trust, Carew House, Dunmere Rd, Beacon Technology ParkBodmin, PL31 2QN UK; 6 Department of Primary Care and Public Health, Imperial College London, London, UK

**Keywords:** Psychosis, Schizophrenia, Severe mental illness, Digital mental health interventions, Mobile applications

## Abstract

**Background:**

Early intervention for psychotic spectrum disorders can improve long-term outcomes, but service availability and quality can vary globally. Mobile apps have the potential to provide personalised and accessible support for people with psychotic disorders via features such as symptom monitoring, medication reminders, and self-management interventions. Existing reviews have provided an overview of such apps and their feasibility but lack a synthesis of their efficacy, safety, and acceptability. Addressing this gap would guide future app designs and facilitate their implementation by informing clinical and policy decisions. The purpose of this systematic review will be to synthesise the evidence about existing mobile apps for psychotic disorders, including their types and features, feasibility of implementation, usability, clinical impact, and safety.

**Methods:**

This protocol has been structured using the PRISMA-P checklist, and the PICOS framework will guide the search strategy. Six electronic databases (PubMed, Embase, CINAHL, Scopus, Web of Science, and PsycInfo) will be searched. Evaluations of mobile apps for psychotic disorders published in English will be included. Reviews will be excluded but their bibliographies will be searched for relevant articles. Two independent reviewers will conduct the title and abstract screening, full-text review, data extraction into a predetermined form, and risk of bias analysis; with any disagreements discussed until consensus at each stage. The risk of bias analysis will be conducted using the Cochrane Collaboration Risk of Bias 2 and Mixed Methods Appraisal Tool. Meta-analyses will summarise data on feasibility, impact, and safety (where applicable), and app characteristics and user experience will be descriptively analysed.

**Discussion:**

While current reviews synthesise information about apps for psychotic disorders, most of them have a narrow focus on specific app types (e.g., monitoring), outcomes (e.g., engagement), or study types. This systematic review will update previous reviews and add a comprehensive synthesis of app features, their safety, and their overall impact. This review will inform future app development and evaluations and facilitate their implementation in clinical services. It will also address the potential negative impacts associated with these apps and propose ways to mitigate them.

**Systematic review registration:**

PROSPERO CRD42024615781.

**Supplementary Information:**

The online version contains supplementary material available at 10.1186/s13643-025-02943-8.

## Background

Psychotic disorders (i.e., schizophrenia spectrum and related disorders) can be severe and debilitating, often associated with high disability and increased premature mortality than the general population [1, 2]. Research suggests that early interventions for psychosis (EIP) can improve mental health and well-being outcomes in the long term, but service availability and quality vary globally. Even in countries such as the UK, which has a relatively robust EIP service, issues such as regional variation in quality, racial inequalities, stigma, and perceived stigma can be significant barriers to seeking and accessing support [3–9]. Mobile technologies are emerging as accessible support for many mental health conditions, including psychotic disorders [10]. Mobile phones can support people with psychotic disorders in a variety of ways, including monitoring, medication adherence, and theory-informed intervention programmes [11, 12]. Previous research suggests that mobile apps can support this population, and several apps have been developed specifically for people with early psychosis or psychotic disorders [13–15]. Despite growing evidence of their impact, there is a lack of implementation of such tools in mental health services, necessitating a comprehensive synthesis of the evidence concerning these apps to ensure the future development of efficacious apps and facilitate their implementation in healthcare services [16, 17]. This review will look to address this gap by summarising the evidence of the feasibility, usability, safety, and impact of mobile apps for psychotic disorders.

Many mobile apps have been developed to address these issues, supporting patients with symptom monitoring, medication reminders or side effect monitoring, or providing psychoeducation or therapeutic interventions. Existing studies suggest that the apps are feasible and generally acceptable, but include mixed evidence of efficacy, with some apps having a significant positive impact on mental health outcomes compared to controls and others resulting in no outcome improvement [18–21]. Previous evaluations also indicate that the apps are generally safe, with few adverse events being related to the studies or apps [18–22], but the clarity and level of detail in reporting is inconsistent, with a significant number of studies not reporting adverse events at all [23].

While several reviews have been published about digital tools for psychosis or schizophrenia-spectrum disorders, they often include general digital technologies without a specific focus on mobile apps (such as videoconferencing or virtual reality) [24–26] or focus only on specific evaluation outcomes (such as engagement or factors impacting implementation) [27–29], study types (such as randomised controlled trials (RCTs) or qualitative studies) [30–33], or types of interventions (such as symptom monitoring) [34–36]. Those reviews that conducted a more comprehensive synthesis found preliminary evidence of feasibility and user-centred design but lacked a focus on efficacy and safety [37, 38]. Additionally, a search on the International Prospective Register of Systematic Reviews (PROSPERO) using the keywords (psychosis OR psychotic OR schizo*) AND (mobile OR digital OR mhealth) found only one ongoing review about mobile apps for psychotic disorders which had a narrow focus on apps for medication adherence. This is a significant gap in the literature as an understanding of the safety and efficacy of the apps is crucial for their implementation in healthcare services.

Given that several apps for psychotic disorders have been developed and evaluated, particularly after 2020 (following the COVID-19 pandemic and the publication of most relevant reviews identified), a systematic review is needed to synthesise their findings and update previous reviews, adding an analysis of previously missed elements such as safety and efficacy. Evidence suggests a marked increase in the development and use of digital mental health tools post-pandemic, further highlighting the need for an updated synthesis [39–42]. Such a review would help inform the design and development of new mobile interventions and facilitate their implementation in existing clinical services to ensure accessible, equitable, and beneficial support for this population.

### Objectives

This review will aim to synthesise the evidence concerning the impact of mobile apps for supporting patients with psychotic disorders. This will be guided by four key objectives: to summarise the types and features of mobile apps for psychotic disorders with a focus on the use of theoretical frameworks (e.g. clinical-psychological models such as Cognitive Behavioural Therapy, behavior-change theories such as the Social Cognitive Theory, or recovery-oriented approaches such as CHIME); to examine the recruitment, retention, and adherence of the apps to analyse their feasibility; to investigate the apps' usability and clinical efficacy; and to summarise their potential negative impacts and safety.

## Methods

This protocol has been structured in line with the Preferred Reporting Items for Systematic reviews and Meta-Analyses Protocols (PRISMA-P) guidelines [43] and the Population, Intervention, Comparison, Outcome, and Study (PICOS; see Table 1) [44, 45] framework helped structure the search strategy. This review protocol was registered with the International Prospective Register of Systematic Reviews (PROSPERO) on 26th November 2024 (ID: CRD42024615781).

**Table 1:** PICOS

Component	Inclusion criteria	Exclusion criteria	MeSH terms	Keywords^a^
Population	People with psychotic disorders	People with any other conditions	Schizophrenia Spectrum and Other Psychotic Disorders	psychosis OR psychotic OR “severe mental” OR “serious mental” OR schizo* OR delusion* OR antipsychotic
Intervention	Mobile apps aiming to monitor or improve outcomes in people with psychotic conditions (including medication adherence and side effect monitoring apps)	Other digital interventions; SMS or social media interventions; apps used only to facilitate remote sessions with clinicians or book appointments; apps where patients are not the end users	Mobile Applications OR Digital Health OR Telemedicine	app OR apps OR “mobile app*” OR “mhealth” OR telemedicine OR “mobile device*” OR smartphone OR “mobile phone” OR “mHealth” OR “mobile health” OR “health app” OR “digital app” OR telepsychology OR “digital intervention” OR “digital mental health intervention” OR “DMHI” OR iphone OR “cell phone*” OR “phone app” OR "digital health"
Comparator	No comparator, standard care (or treatment as usual), waitlist controls, and face-to-face intervention (separate meta-analyses for each comparator if feasible)	N/A	N/A	N/A
Outcomes	Impact on mental health (measured by improvement in psychotic symptoms, wellbeing, quality of life, functioning, etc.), recruitment rates, adherence, retention, usability, user experience, safety (measured using adverse event monitoring)	N/A (specific outcomes not required for inclusion as long as the studies evaluate the app)	N/A	Evaluat* OR effective* OR impact OR improve* OR efficacy OR study OR trial* OR studies OR “mental health” OR wellbeing OR “well-being” OR symptom* OR usability OR “user experience” OR “user satisfaction” OR acceptability OR safety OR “adverse event*” OR engagement OR adherence
Study	All studies that evaluate a mobile app for psychosis will be included with no restrictions on study design.	Reviews, protocols, design and development studies, conference abstracts, posters, studies not published in English, papers published before 2008	N/A	N/A

^a^Searched in Title/Abstract unless otherwise specified

### Search strategy

The search will be conducted in six databases: PubMed, CINAHL, Embase, Scopus, Web of Science, and PsycInfo. A preliminary literature search and previous reviews on digital interventions for schizophrenia and other psychotic spectrum disorders, along with the PICOS framework, guided the selection of relevant keywords and MeSH (Medical Subject Headings) terms. They were grouped into two themes and structured as follows: psychosis (MeSH OR Keywords) AND mobile apps (MeSH OR Keywords) AND evaluation outcomes (MeSH OR Keywords) (see Table 1). In addition to the database search using the above keywords, the bibliographies of relevant reviews will be manually screened to avoid missing relevant articles.

### Eligibility criteria

#### Inclusion criteria

The review will include all studies published in peer-reviewed journals since 2008 (coinciding with the launch of the first app store) that evaluate mobile apps for people with a clinical diagnosis of a psychotic spectrum disorder (such as schizophrenia or schizoaffective disorder, or other psychotic disorders). No restrictions will be placed on study design or the age of the target population.

#### Exclusion criteria

Studies that do not evaluate one or more relevant mobile apps (e.g., protocols, reviews, descriptions of design or development) will be excluded. Studies evaluating SMS or social media interventions using existing apps (e.g., WhatsApp, Facebook, etc.), apps used purely to facilitate remote sessions with clinicians (including appointment-booking apps for in-person therapy), or apps designed only for clinicians or researchers (i.e., where patients are not the end users) will also be excluded. Finally, studies not published in English and those with no full-text availability will be excluded from this review.

### Screening

All references identified in the literature search will be downloaded and stored in the citation management software Endnote 21, which will be used for deduplication and automatic keyword-based searches to exclude ineligible studies over multiple passes (recorded in an Appendix). The references will then be imported into the online systematic review platform, Rayyan, which will be used to capture any remaining duplicates. Two independent reviewers will conduct title and abstract screening of the references, followed by full-text screening, at which stage the reasons for exclusion will be recorded. Any difference of opinion during screening will be discussed until consensus and a third reviewer will adjudicate if an agreement cannot be reached. To ensure replicability and transparency of each stage of the process, the details will be recorded in a PRISMA flow diagram.

### Data extraction

The two reviewers will independently examine the full-texts of all included papers and extract data into a predetermined form (Table 2). As with the screening, any disagreements will be discussed until consensus and a third reviewer will adjudicate if necessary.

**Table 2:** Data extraction items

**Study characteristics**	Author
	Year
	Title
	Country
**App characteristics**	Name of the app
	Purpose of the app ​​(broad anticipated categories: medication adherence/reminders; monitoring; targeting specific aspects of psychosis; multiple)
	Key features
	The use of theoretical framework (if applicable), categorised as follows: • Clinical (e.g., Cognitive Behaviour Theory, Acceptance and Commitment Theory, etc.) • Behaviour change (e.g., Social Cognitive Theory, Behaviour Change Wheel framework, Health Belief Model, etc.) • Recovery-focused (e.g., CHIME (Connectedness, Hope, Identity, Meaning, and Empowerment) framework) • Other (i.e., any framework used that does not fit into the above categories)
	Data sharing
	Who can access the data?
	Available in the marketplace? (Yes/No)
	Android/iOS?
**Evaluation**	Study design
	Sample size (at baseline)
	Sample demographics
	Study duration
	Primary aim
	Recruitment
	Retention
	Engagement
	Usability; user experience • Y/N • Scales used (e.g., System Usability Scale [46]) • Findings
	Clinical outcomes • Outcome measures (e.g., wellbeing, depression/anxiety symptoms, psychotic symptoms, relapse prediction) • Scales used (e.g., Brief Psychiatric Rating Scale, Positive and Negative Syndrome Scale) • Findings
	Adverse events
	Cost-effectiveness reported? (Y/N)

### Quality appraisal and risk of bias analysis

Two reviewers will independently conduct a risk of bias analysis for all the included studies, with disagreements discussed and resolved by a senior researcher if necessary. The RCTs will be assessed using the Cochrane Collaboration Risk of Bias 2 tool (RoB2) [47, 48], while the risk of bias of other study designs will be analysed using the Mixed Methods Appraisal Tool (MMAT) [49]. Figures or tables will summarise the risk of bias findings and the analysis data will be recorded in the data extraction spreadsheet.

### Data analysis and synthesis

#### Meta-analyses

While a variety of study types, aims, and reported outcomes are expected, we plan to conduct a meta-analysis to analyse the feasibility of mobile apps for psychotic disorders by conducting proportional meta-analyses on pooled data related to the rates of recruitment, retention, and engagement (where applicable). If studies reporting efficacy data have sufficiently homogeneous aims and outcomes, they will be meta-analysed to determine overall efficacy. For example, studies of apps aiming to increase medication adherence and those including interventions to directly improve symptoms would have different aims and outcomes and would therefore be analysed separately. We also plan to conduct a meta-analysis for the safety of such interventions using adverse event data, if consistently reported. The analyses will use a random-effects model, statistical heterogeneity will be measured using a Tau-squared test and the *I*^*2*^ measure, and the Egger’s test will be used to assess publication bias. To account for the different scales that might be used to assess impact on mental health outcomes, the meta-analysis will use the standardised mean difference [50].

#### Other analyses

Any data that cannot be meta-analysed, including information about the types and features of the apps, their usability and user experience, and mental health or other clinical outcomes evaluated via non-RCT designs will be summarised using a narrative synthesis methodology [51], chosen as it allows the integration of diverse qualitative and quantitative research findings.

## Results

The systematic review is expected to be completed and submitted for publication in a peer-reviewed journal by June 2026.

## Discussion

This systematic review will add to the literature by summarising and synthesising the evidence of real-world impact of mobile apps for psychotic disorders. This review will serve as an update to previous reviews of apps for psychotic disorders (e.g., [26, 31, 37, 38], and add a comprehensive summary of app features, their theoretical basis, and their safety. By providing such a synthesis, we aim to provide a valuable overview to inform the decisions of future app developers, healthcare providers, and policymakers around the implementation of such apps in existing clinical services. Our meta-analysis will provide a valuable summary of the feasibility and clinical impact of these apps, considering their potential negative impacts. The descriptive analysis and qualitative synthesis will provide insight into their characteristics, usability and acceptability.

Our planned review has a few potential limitations. First, as we seek to synthesise a broad range of literature, we anticipate substantial heterogeneity in intervention types, study designs, sample sizes, durations, and outcomes. This may limit the generalisability of our meta-analysis findings. To mitigate this, the results will also be narratively synthesised, enabling insights where meta-analyses are not feasible or meaningful. Additionally, due to the language capabilities of the research team, our review is limited to studies published in English, which may skew our data towards English-speaking countries and neglect relevant research in other parts of the world.

By combining qualitative and quantitative synthesis methods to summarise the evidence regarding mobile apps for psychotic disorders, this review will provide a comprehensive overview of the usability, acceptability, impact, and safety of these apps. Such a review will guide the future development of efficacious, safe, and acceptable apps for psychotic disorders and inform decisions around their clinical implementation.

## Supplementary Information

Additional file 1: PRISMA checklist.

Additional file 2: Sample search strings.

## Data Availability

All the data used in this article are from publicly available sources, which have been cited where applicable.
