# Supplementary material for: Mobile apps for psychotic disorders: a systematic review protocol
Source: Syst Rev. 2026 Jan 9;15:46. doi: 10.1186/s13643-025-02943-8 (PMC12882349; doi:10.1186/s13643-025-02943-8)
Supplement: Supplementary file 2 — Additional file 2: Sample search strings. [file 13643_2025_2943_MOESM2_ESM.docx]

## Appendix 2. Sample search strings

| **Database** | **Search string** | **Results** |
| --- | --- | --- |
| PubMed | ("Mobile Applications"[MeSH Terms] OR "Digital Health"[MeSH Terms] OR "Telemedicine"[MeSH Terms] OR ("app"[Title/Abstract] OR "apps"[Title/Abstract] OR "mobile app*"[Title/Abstract] OR "mHealth"[Title/Abstract] OR "Telemedicine"[Title/Abstract] OR "mobile device*"[Title/Abstract] OR "smartphone"[Title/Abstract] OR "mobile phone"[Title/Abstract] OR "mHealth"[Title/Abstract] OR "mobile health"[Title/Abstract] OR "health app"[Title/Abstract] OR "digital app"[Title/Abstract] OR "telepsychology"[Title/Abstract] OR "digital intervention"[Title/Abstract] OR "digital mental health intervention"[Title/Abstract] OR "DMHI"[Title/Abstract] OR "iphone"[Title/Abstract] OR "cell phone*"[Title/Abstract] OR "phone app"[Title/Abstract] OR "Digital Health"[Title/Abstract])) AND ("evaluat*"[Title/Abstract] OR "effective*"[Title/Abstract] OR "impact"[Title/Abstract] OR "improve*"[Title/Abstract] OR "efficacy"[Title/Abstract] OR "study"[Title/Abstract] OR "trial*"[Title/Abstract] OR "studies"[Title/Abstract] OR "mental health"[Title/Abstract] OR "wellbeing"[Title/Abstract] OR "well-being"[Title/Abstract] OR "symptom*"[Title/Abstract] OR "usability"[Title/Abstract] OR "user experience"[Title/Abstract] OR "user satisfaction"[Title/Abstract] OR "acceptability"[Title/Abstract] OR "safety"[Title/Abstract] OR "adverse event*"[Title/Abstract] OR "engagement"[Title/Abstract] OR "adherence"[Title/Abstract]) AND ("Schizophrenia Spectrum and Other Psychotic Disorders"[MeSH Terms] OR ("psychosis"[Title/Abstract] OR "psychotic"[Title/Abstract] OR "severe mental"[Title/Abstract] OR "serious mental"[Title/Abstract] OR "schizo*"[Title/Abstract] OR "delusion*"[Title/Abstract] OR "antipsychotic"[Title/Abstract])) | 1306 |
| Scopus | ( TITLE-ABS-KEY ( psychosis OR psychotic OR "severe mental" OR "serious mental" OR schizo* OR delusion* OR antipsychotic ) AND TITLE-ABS-KEY ( pp OR apps OR "mobile app*" OR "mhealth" OR telemedicine OR "mobile device*" OR smartphone OR "mobile phone*" OR "mHealth" OR "mobile health" OR "health app" OR "digital app" OR telepsychology OR "digital intervention*" OR "digital mental health intervention*" OR "DMHI" OR iphone* OR "cell phone*" OR "phone app*" OR "Digital Health" ) AND TITLE-ABS-KEY ( evaluat* OR effective* OR impact OR improve* OR efficacy OR study OR trial* OR studies OR "mental health" OR wellbeing OR "well-being" OR symptom* OR usability OR "user experience" OR "user satisfaction" OR acceptability OR safety OR "adverse event*" OR engagement OR adherence ) ) | 2679 |
| Web of Science | TS=(psychosis OR psychotic OR "severe mental" OR "serious mental" OR schizo* OR antipsychotic OR delusion*) AND TS=(app OR apps OR "mobile app*" OR telemedicine OR "mobile device*" OR smartphone OR "mobile phone*" OR "mHealth" OR "mobile health" OR "health app" OR "digital app" OR telepsychology OR "digital intervention*" OR "digital mental health intervention*" OR "DMHI" OR iphone* OR "cell phone*" OR "phone app*" OR "digital health") AND TS=(Evaluat* OR effective* OR impact OR improve* OR efficacy OR study OR trial* OR studies OR "mental health" OR wellbeing OR "well-being" OR symptom* OR usability OR "user experience" OR "user satisfaction" OR acceptability OR safety OR "adverse event*" OR engagement OR adherence) | 1475 |
| CINAHL | ( TI ( Evaluat* OR effective* OR impact OR improve* OR efficacy OR study OR trial* OR studies OR "mental health" OR wellbeing OR "well-being" OR symptom* OR usability OR "user experience" OR "user satisfaction" OR acceptability OR safety OR "adverse event*" OR engagement OR adherence ) OR AB ( Evaluat* OR effective* OR impact OR improve* OR efficacy OR study OR trial* OR studies OR "mental health" OR wellbeing OR "well-being" OR symptom* OR usability OR "user experience" OR "user satisfaction" OR acceptability OR safety OR "adverse event*" OR engagement OR adherence ) ) AND ( ( (MH "Digital Health+") OR (MH "Telehealth+") OR (MH "Mobile Applications") ) OR TI ( app OR apps OR "mobile app*" OR "mhealth" OR telemedicine OR "mobile device*" OR smartphone OR "mobile phone*" OR "mHealth" OR "mobile health" OR "health app" OR "digital app" OR telepsychology OR "digital intervention*" OR "digital mental health intervention*" OR "DMHI" OR iphone* OR "cell phone*" OR "phone app*" OR "Digital Health" ) OR AB ( app OR apps OR "mobile app*" OR "mhealth" OR telemedicine OR "mobile device*" OR smartphone OR "mobile phone*" OR "mHealth" OR "mobile health" OR "health app" OR "digital app" OR telepsychology OR "digital intervention*" OR "digital mental health intervention*" OR "DMHI" OR iphone* OR "cell phone*" OR "phone app*" OR "Digital Health" ) ) AND ( (MH "Psychotic Disorders+") OR TI ( psychosis OR psychotic OR "severe mental" OR "serious mental" OR schizo* OR antipsychotic OR delusion* ) OR AB ( psychosis OR psychotic OR "severe mental" OR "serious mental" OR schizo* OR antipsychotic OR delusion* ) ) | 1563 |
| PsycInfo | ((app or apps or "mobile app*" or "mhealth" or telemedicine or "mobile device*" or smartphone or "mobile phone*" or "mHealth" or "mobile health" or "health app" or "digital app" or telepsychology or "digital intervention*" or "digital mental health intervention*" or "DMHI" or iphone* or "cell phone*" or "phone app*" or "Digital Health").ti. or (app or apps or "mobile app*" or "mhealth" or telemedicine or "mobile device*" or smartphone or "mobile phone*" or "mHealth" or "mobile health" or "health app" or "digital app" or telepsychology or "digital intervention*" or "digital mental health intervention*" or "DMHI" or iphone* or "cell phone*" or "phone app*" or "Digital Health").ab. or exp Electronic Health Services/ or exp Telemedicine/ or exp Mobile Applications/) and ((evaluat* or effective* or impact or improve* or efficacy or study or trial* or studies or "mental health" or wellbeing or "well-being" or symptom* or usability or "user experience" or "user satisfaction" or acceptability or safety or "adverse event*" or engagement or adherence).ti. or (evaluat* or effective* or impact or improve* or efficacy or study or trial* or studies or "mental health" or wellbeing or "well-being" or symptom* or usability or "user experience" or "user satisfaction" or acceptability or safety or "adverse event*" or engagement or adherence).ab.) and ((psychosis or psychotic or "severe mental" or "serious mental" or schizo* or antipsychotic or delusion*).ti. or (psychosis or psychotic or "severe mental" or "serious mental" or schizo* or antipsychotic or delusion*).ab. or exp Psychosis/) | 1266 |
| Embase | (exp mobile application/ or exp telehealth/ or exp digital health/ or (app or apps or "mobile app*" or "mhealth" or telemedicine or "mobile device*" or smartphone or "mobile phone*" or "mHealth" or "mobile health" or "health app" or "digital app" or telepsychology or "digital intervention*" or "digital mental health intervention*" or "DMHI" or iphone* or "cell phone*" or "phone app*" or "Digital Health").ti. or (app or apps or "mobile app*" or "mhealth" or telemedicine or "mobile device*" or smartphone or "mobile phone*" or "mHealth" or "mobile health" or "health app" or "digital app" or telepsychology or "digital intervention*" or "digital mental health intervention*" or "DMHI" or iphone* or "cell phone*" or "phone app*" or "Digital Health").ab.) and ((evaluat* or effective* or impact or improve* or efficacy or study or trial* or studies or "mental health" or wellbeing or "well-being" or symptom* or usability or "user experience" or "user satisfaction" or acceptability or safety or "adverse event*" or engagement or adherence).ti. or (evaluat* or effective* or impact or improve* or efficacy or study or trial* or studies or "mental health" or wellbeing or "well-being" or symptom* or usability or "user experience" or "user satisfaction" or acceptability or safety or "adverse event*" or engagement or adherence).ab.) and ((psychosis or psychotic or "severe mental" or "serious mental" or schizo* or antipsychotic or delusion*).ti. or (psychosis or psychotic or "severe mental" or "serious mental" or schizo* or antipsychotic or delusion*).ab. or exp psychosis/ or exp schizophrenia spectrum disorder/) | 2487 |
| Total | N/A | 10776 |
